# Supplementary material for: Positional proteomics reveals differences in N‐terminal proteoform stability
Source: Mol Syst Biol. 2016 Feb 18;12(2):858. doi: 10.15252/msb.20156662 (PMC4770386; doi:10.15252/msb.20156662)
Supplement: Supplementary file 1 — Expanded View Figures PDF [file MSB-12-858-s001.pdf]

Expanded View Figures

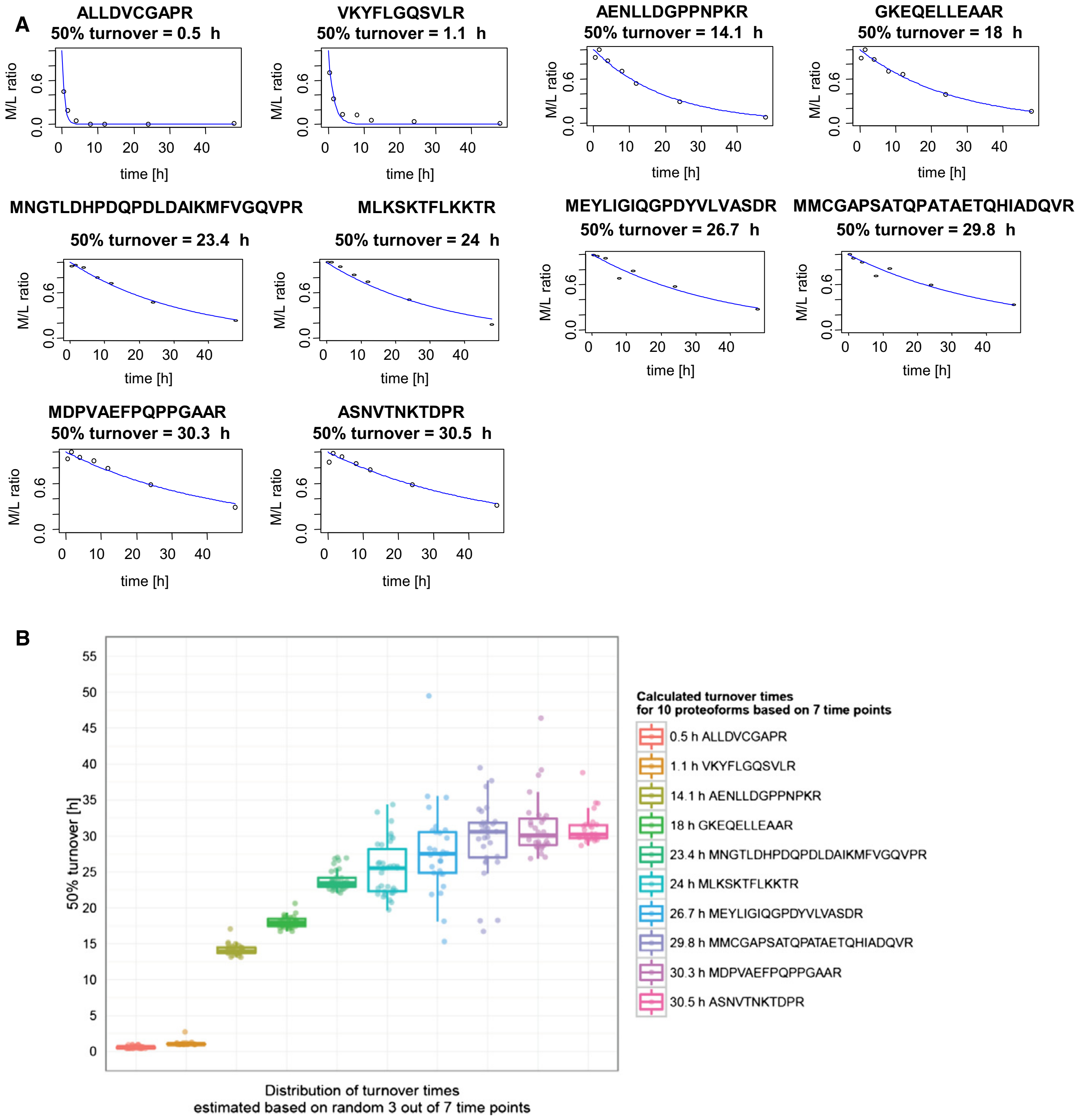

Figure EV1. Turnover time calculations using a subset of available data points.

A Representative proteoforms (10) identified in seven time points displaying a large variety of turnover rates.

B For each peptide, all possible combinations of 3 random points were fitted with an exponential model and used for calculating 50% turnover time. Next, models with R^2 coefficient below 0.8 were rejected. The remaining valid models were grouped per peptide and represented as box plots.

 © 2016 The Authors

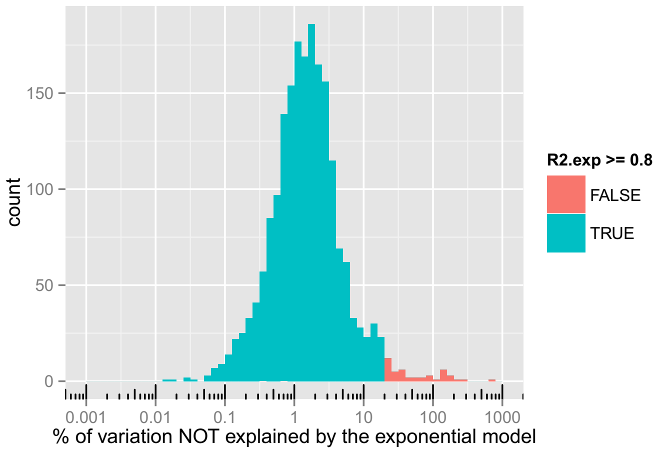

Figure EV2. For each proteoform quantified in at least three time points (1,972), variation of data unexplained by the exponential model was calculated as (1 − R^2) and represented in %.
The distribution of the unexplained variations is tightly centred around 2% (corresponding to a median R^2 = 0.98) while only a minority of exponential models fitted insufficiently (R^2 < 0.8) and were rejected.

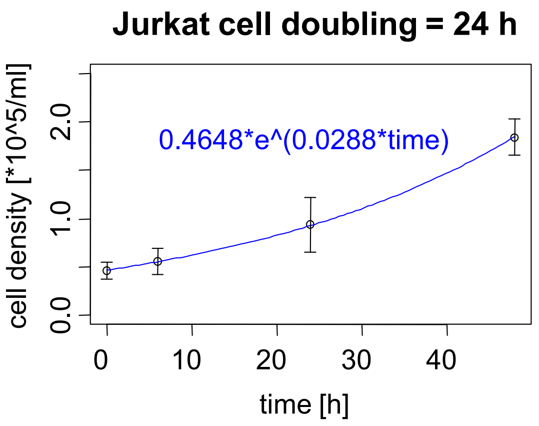

Figure EV3. Experimental assignment of Jurkat cell doubling time.
Density of cell culture was monitored in triplicate over the course of 48 h and fitted with an exponential model using the Doubling Time Online Calculator (http://www.doubling-time.com/compute.php). Cell doubling time was calculated from the assigned growth rate (0.0288) as follows: ln(2)/0.0288 and represented in hours.

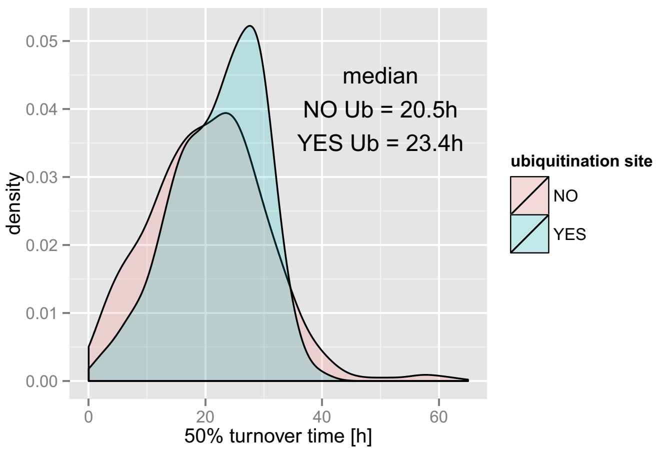

Figure EV4. Distribution of turnover values for lysine containing ubiquitinated (N = 348) and non-ubiquitinated dbTIS indicative N-termini (N = 388).

© 2016 The Authors

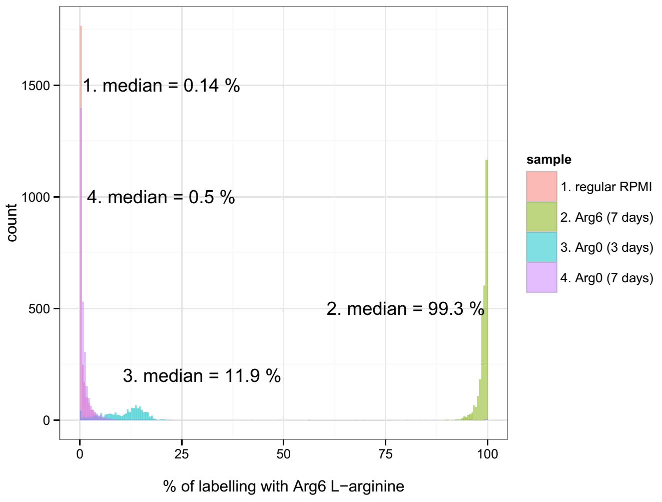

Figure EV5. SILAC labelling of Jurkat cells.
Jurkat cells cultured in regular RPMI medium were transferred to RPMI SILAC medium containing Arg^6 L-arginine and cultured for 7 days. Subsequently, cells were transferred to a RPMI SILAC medium containing Arg^0 L-arginine for next 7 days, to achieve complete unlabelling. The isotope replacement was monitored by quantifying the identified MS spectra using Mascot Distiller.

© 2016 The Authors
