# Supplementary figures and images for: Positional proteomics reveals differences in N‐terminal proteoform stability
Source: Mol Syst Biol. 2016 Feb 18;12(2):858. doi: 10.15252/msb.20156662 (PMC4770386; doi:10.15252/msb.20156662)

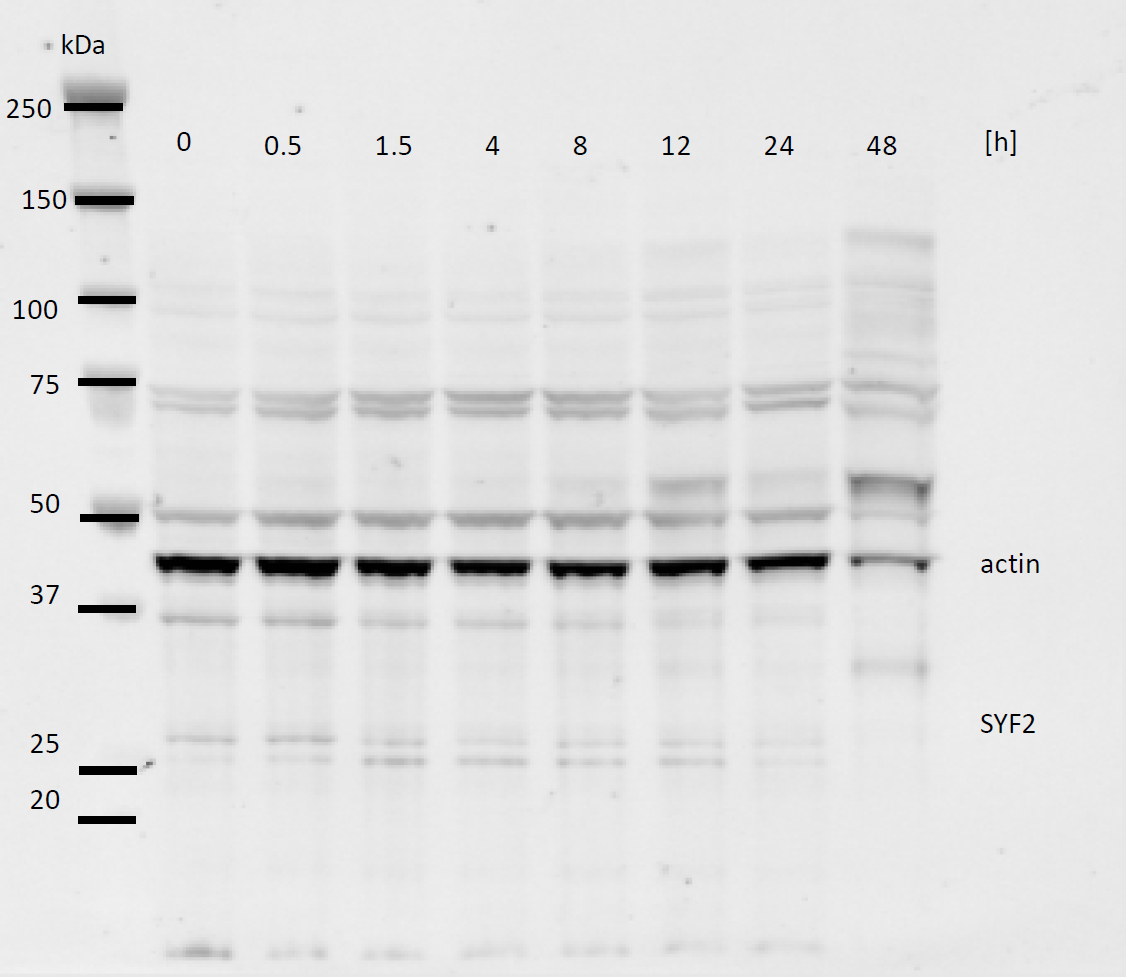

Supplement: Supplementary file 5 — Source Data for Figure 8A [file MSB-12-858-s004.zip › Figure8-panelA-beta-actin.jpg]

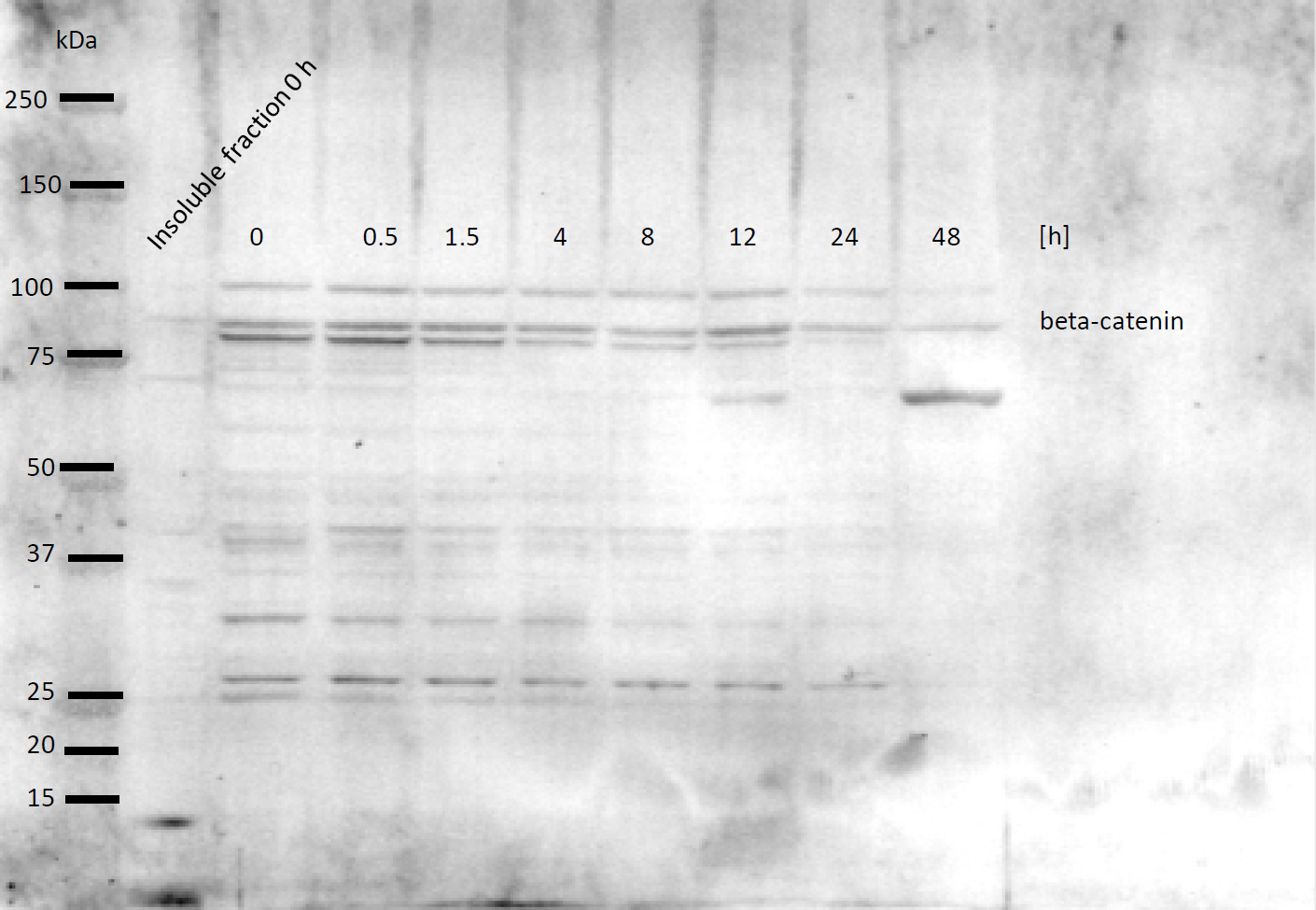

Supplement: Supplementary file 5 — Source Data for Figure 8A [file MSB-12-858-s004.zip › Figure8-panelA-beta-catenin.jpg]

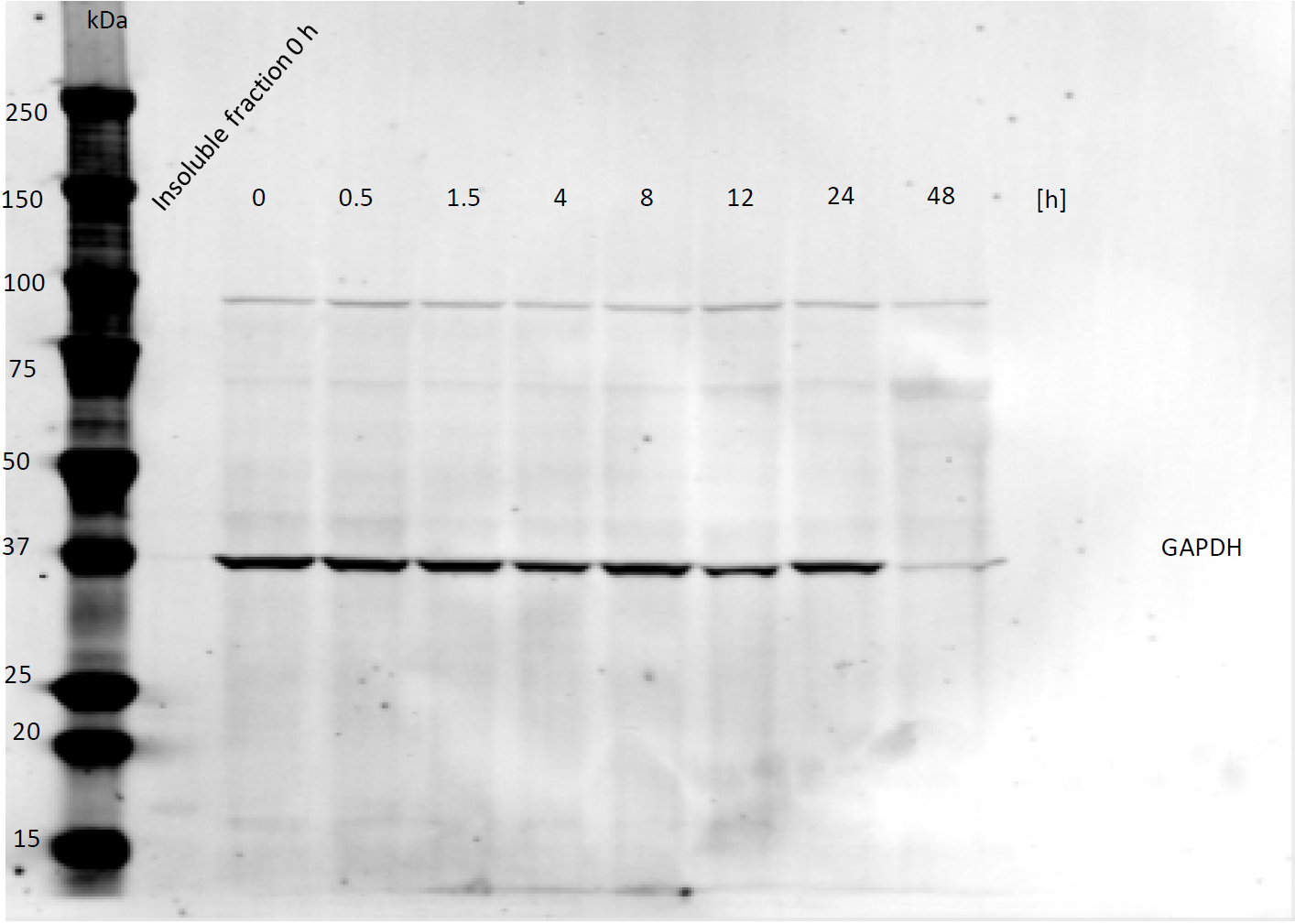

Supplement: Supplementary file 5 — Source Data for Figure 8A [file MSB-12-858-s004.zip › Figure8-panelA-GAPDH.jpg]

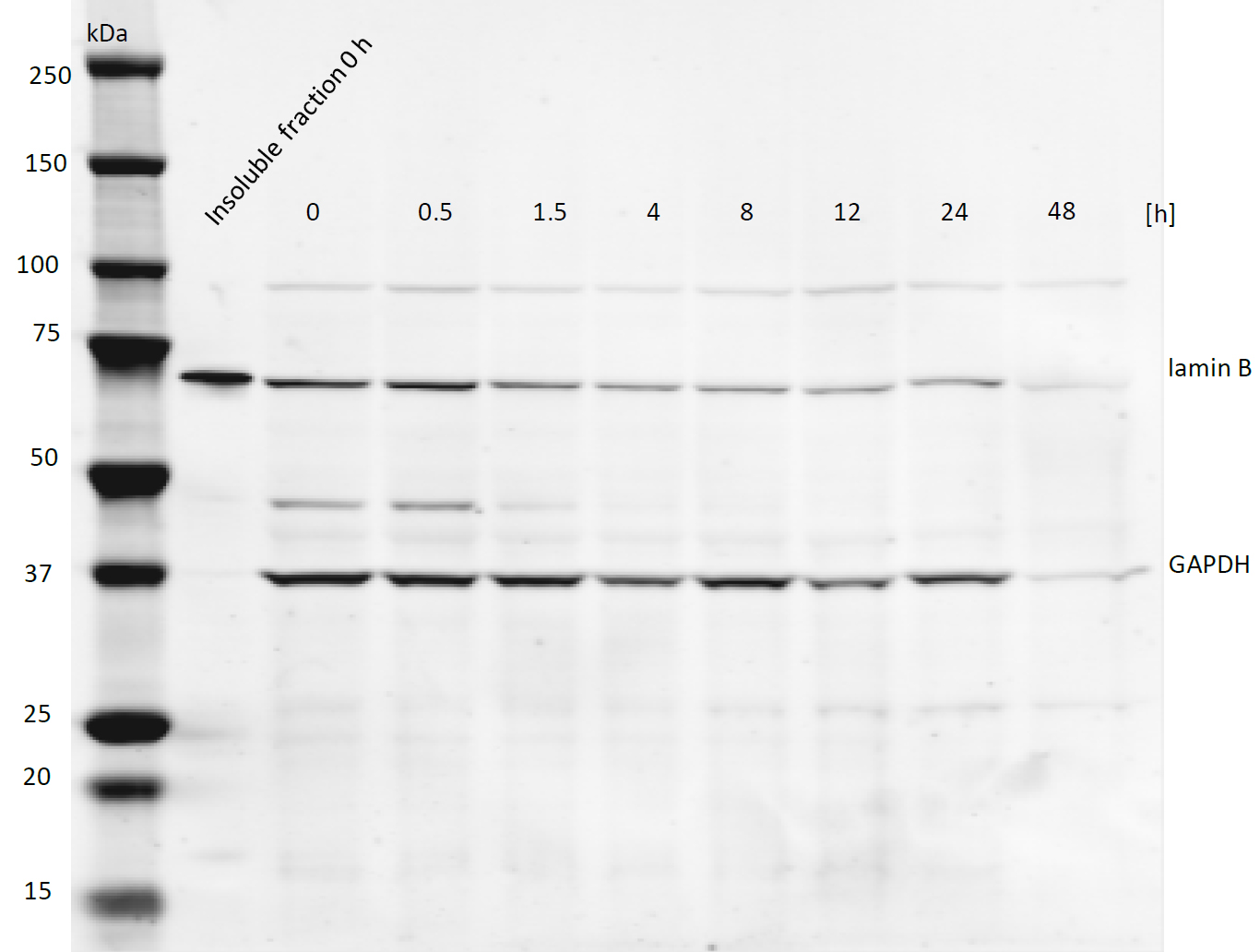

Supplement: Supplementary file 5 — Source Data for Figure 8A [file MSB-12-858-s004.zip › Figure8-panelA-lamin-B.jpg]

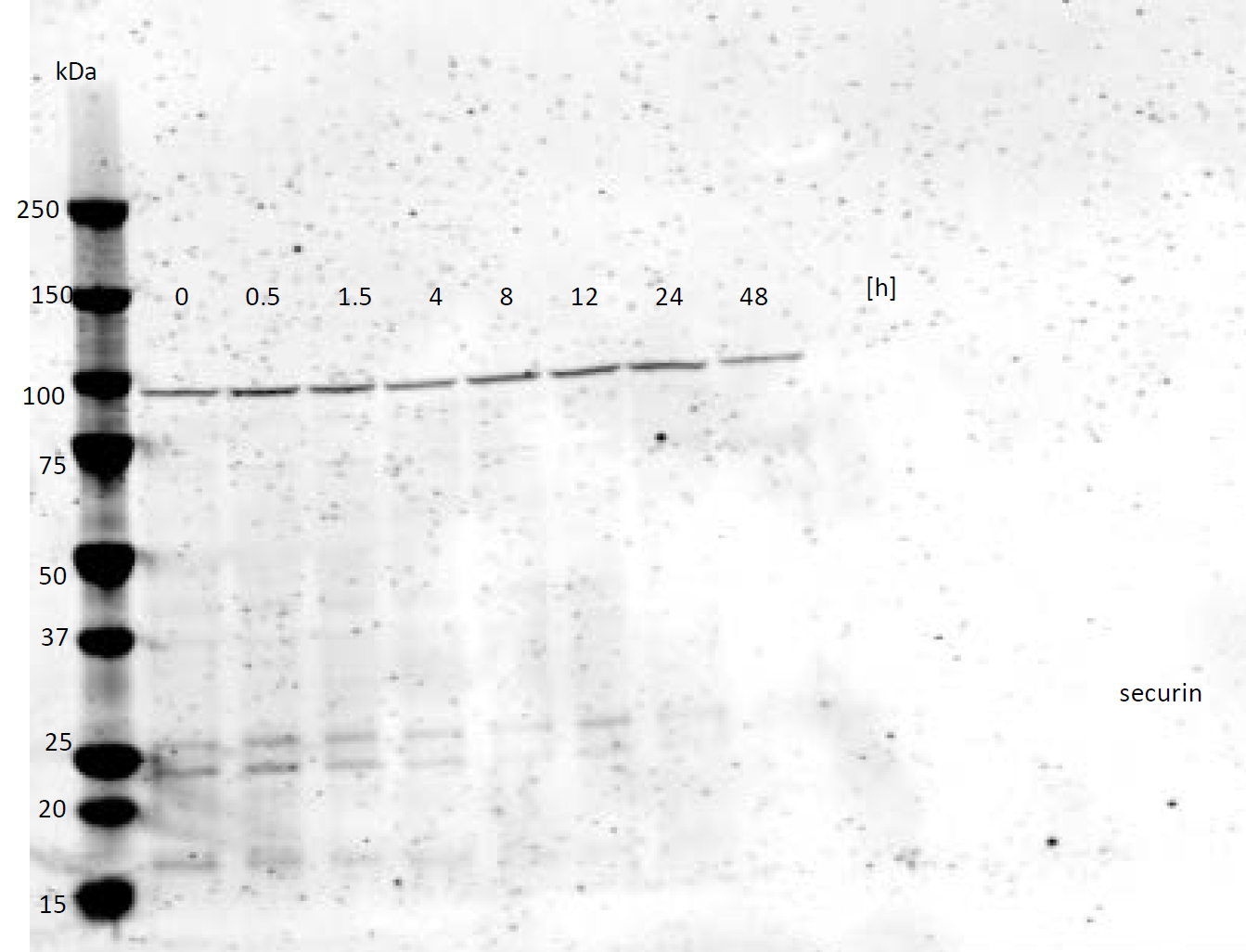

Supplement: Supplementary file 5 — Source Data for Figure 8A [file MSB-12-858-s004.zip › Figure8-panelA-securin.jpg]

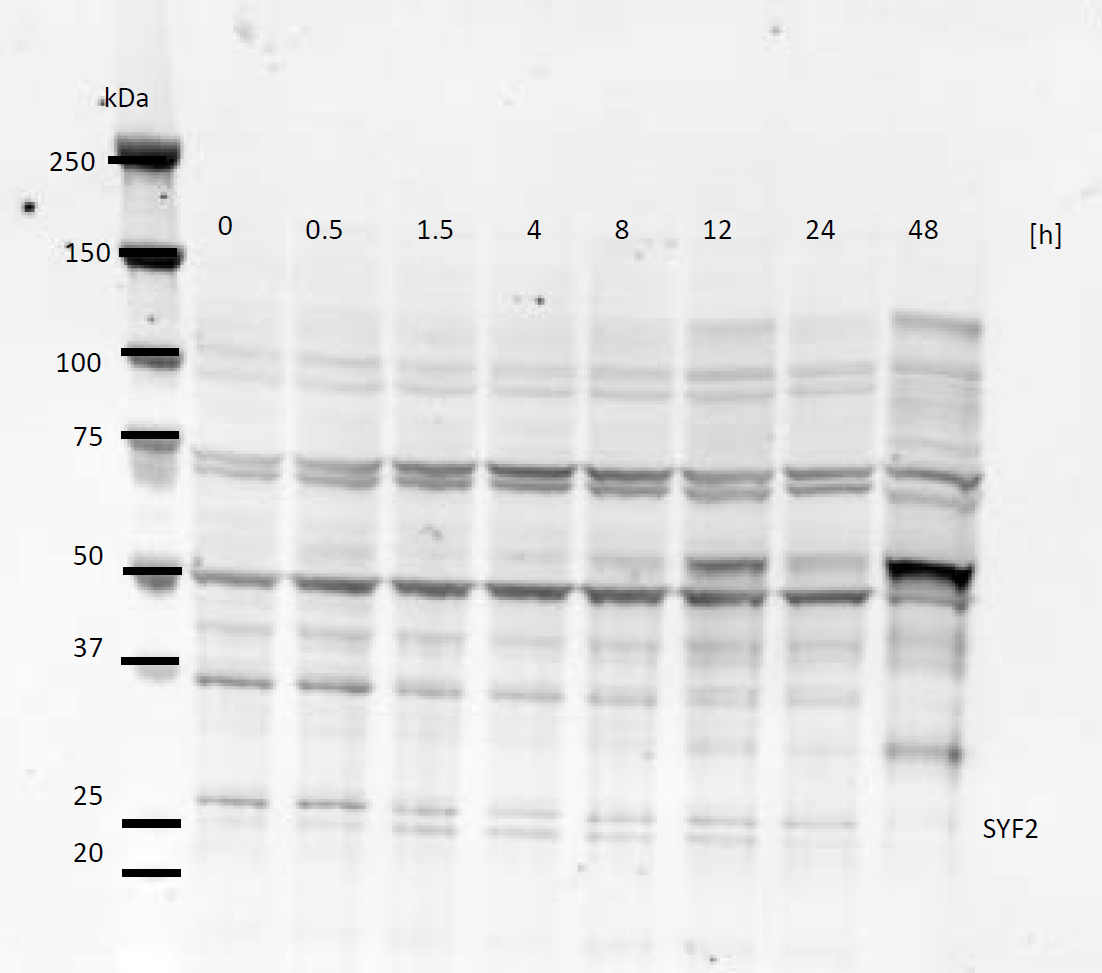

Supplement: Supplementary file 5 — Source Data for Figure 8A [file MSB-12-858-s004.zip › Figure8-panelA-SYF2.jpg]

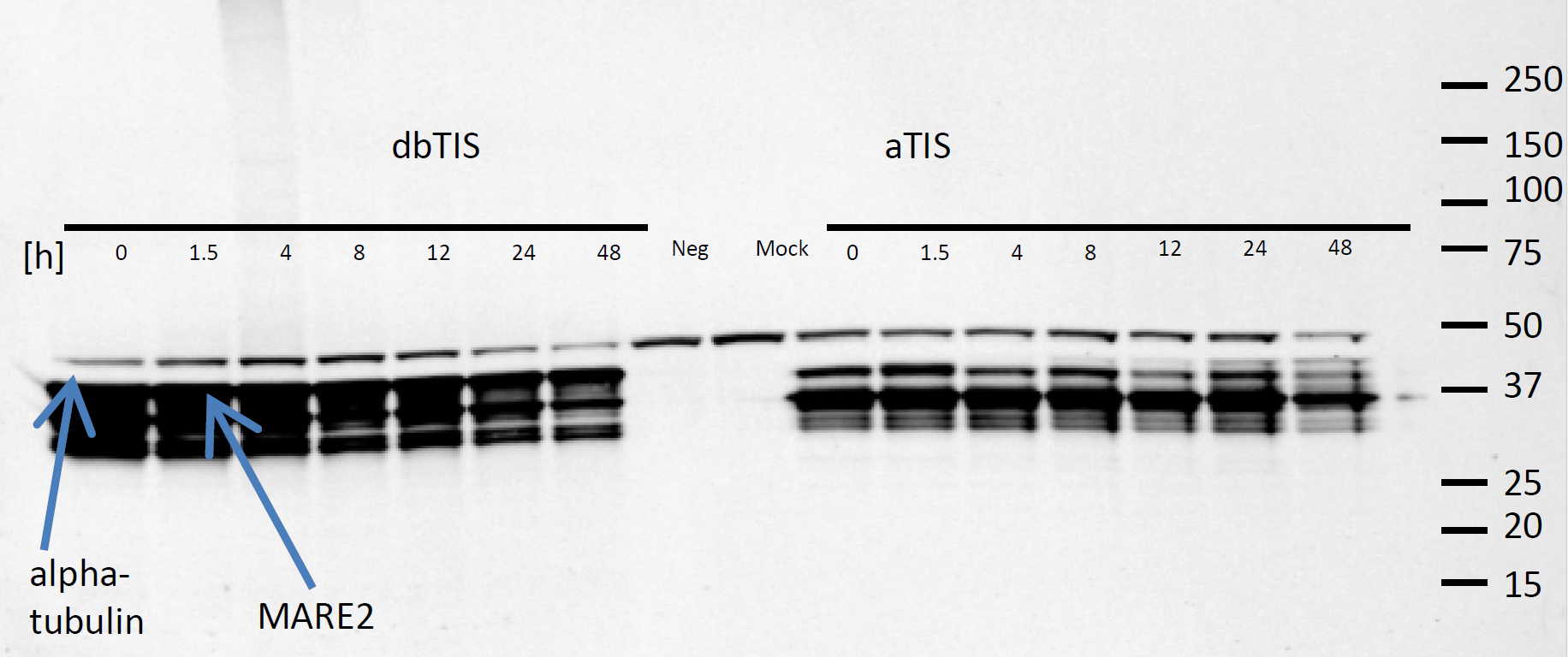

Supplement: Supplementary file 6 — Source Data for Figure 8B [file MSB-12-858-s005.zip › Figure8-panelB-alpha-tubulin.jpg]

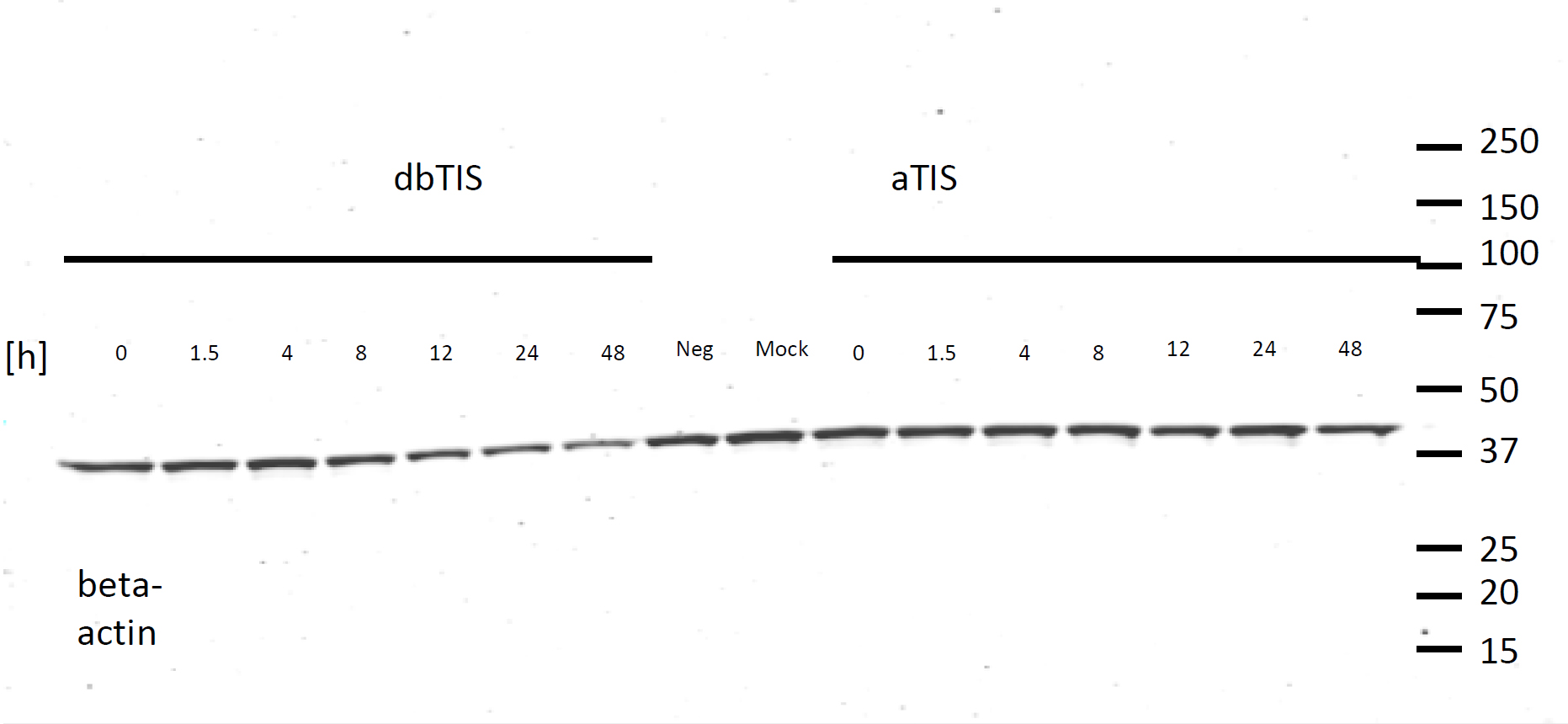

Supplement: Supplementary file 6 — Source Data for Figure 8B [file MSB-12-858-s005.zip › Figure8-panelB-beta-actin.jpg]

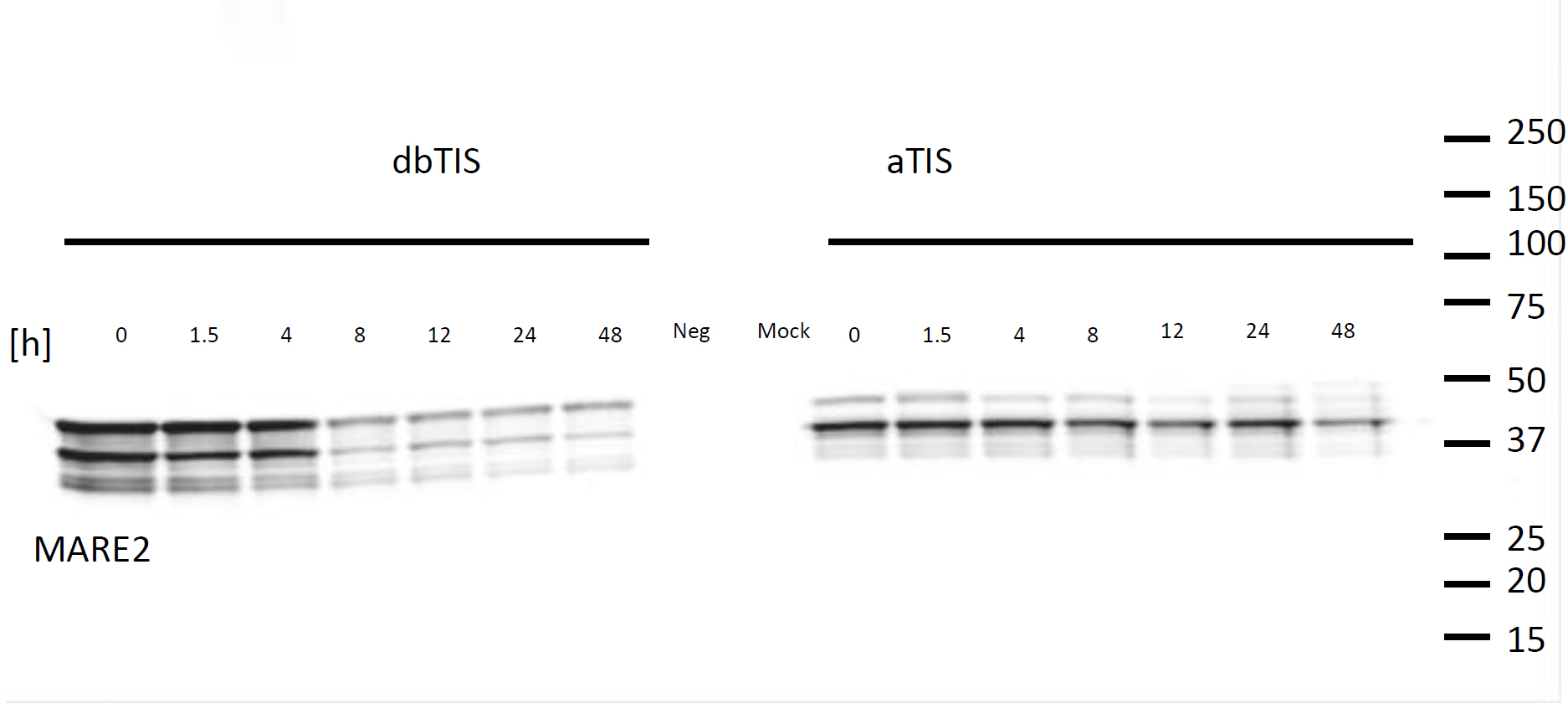

Supplement: Supplementary file 6 — Source Data for Figure 8B [file MSB-12-858-s005.zip › Figure8-panelB-MARE2-V5.jpg]

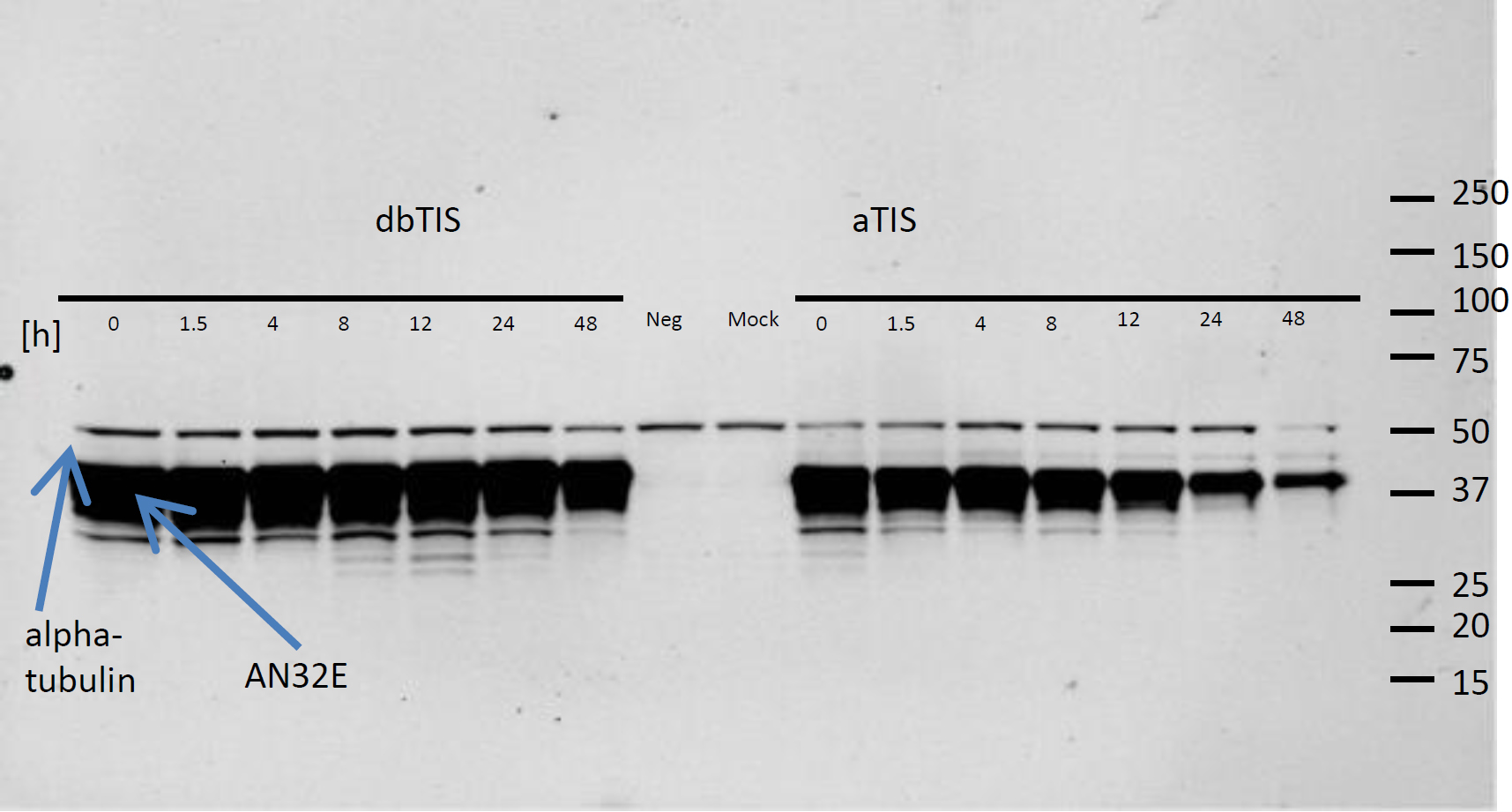

Supplement: Supplementary file 7 — Source Data for Figure 8C [file MSB-12-858-s006.zip › Figure8-panelC-alpha-tubulin.jpg]

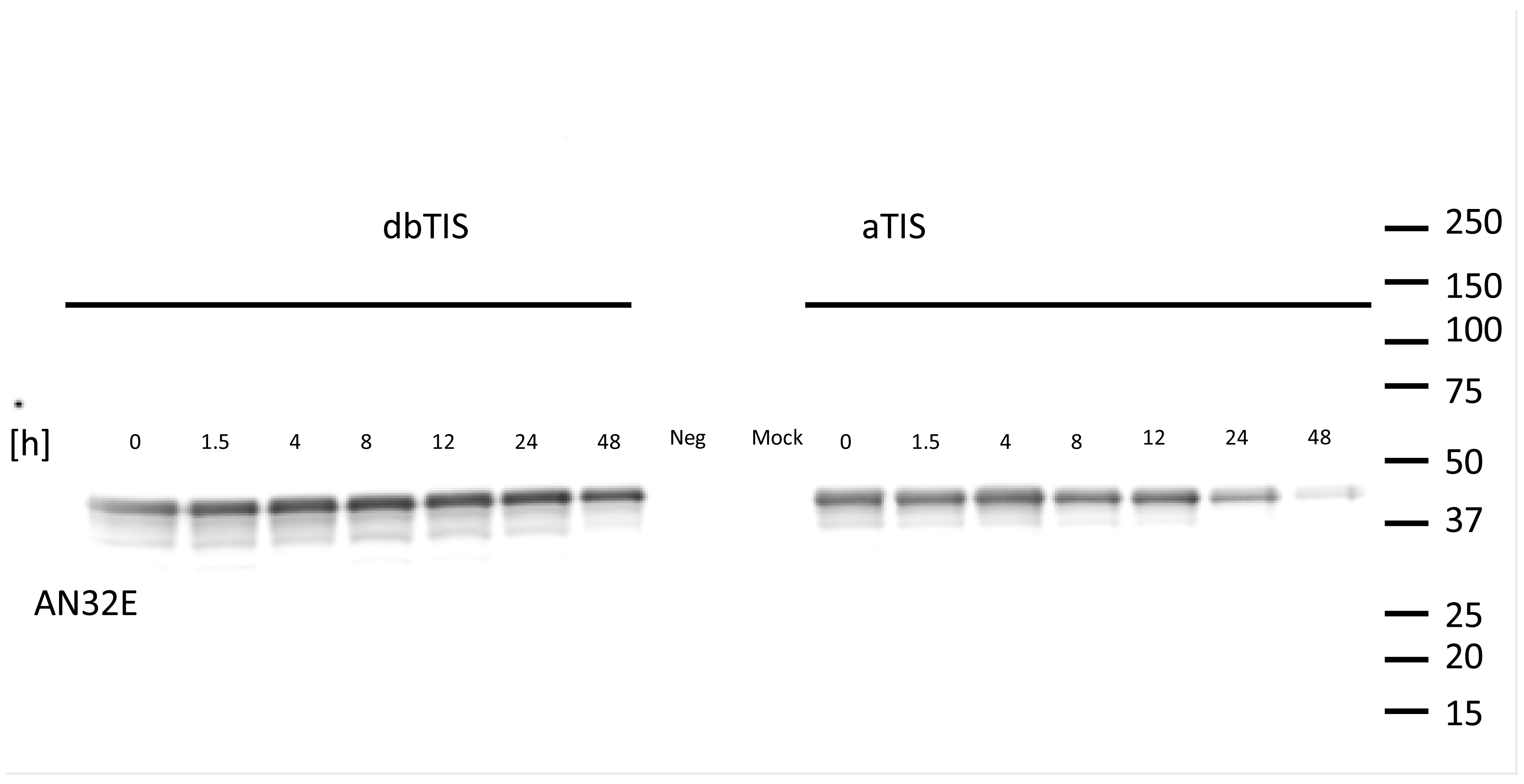

Supplement: Supplementary file 7 — Source Data for Figure 8C [file MSB-12-858-s006.zip › Figure8-panelC-AN32E-V5.jpg]

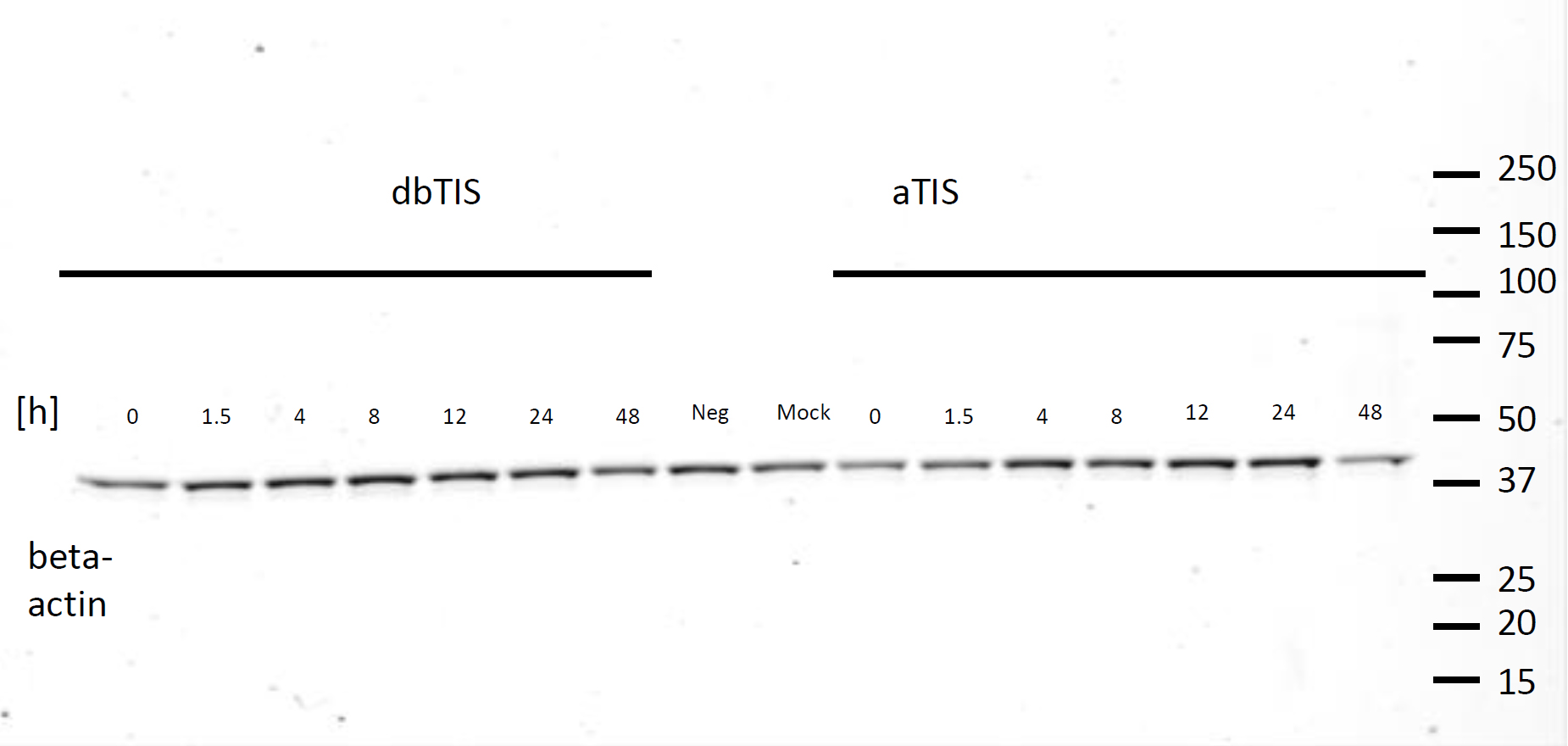

Supplement: Supplementary file 7 — Source Data for Figure 8C [file MSB-12-858-s006.zip › Figure8-panelC-beta-actin.jpg]
